# Tricuspid Regurgitation-Induced Shunt Reversal Leading to Paradoxical Embolic Stroke

**DOI:** 10.1016/j.case.2025.10.004

**Published:** 2025-11-22

**Authors:** Cheng-Hao Jacky Chen, Ujwal Aluru, Ashish Sakharpe, Sri Varsha Pulijal

**Affiliations:** Augusta University, Medical College of Georgia, Evans, Georgia

**Keywords:** Traumatic tricuspid injury, Patent foramen ovale, Fat embolism, Transesophageal echocardiography

## Abstract

• TV injuries are rare but can be clinically challenging.
• Echocardiography is a valuable tool in diagnosing cardiac injuries.
• Significant TR can cause right-to-left flow in an interatrial shunt.
• Surgical intervention may halt progression to right heart failure.

## Graphical abstract

**Figure:**
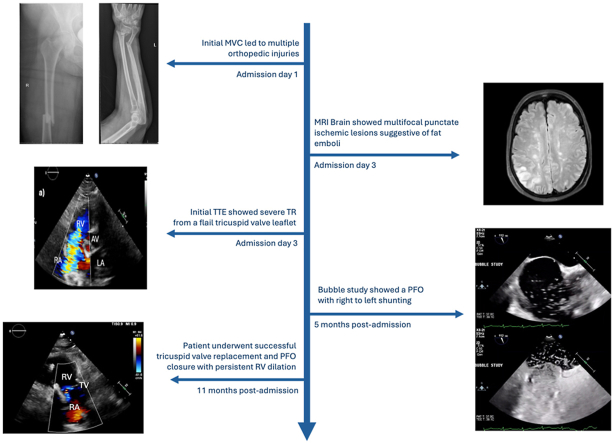

## Introduction

Blunt cardiac trauma rarely occurs in isolation and is typically associated with other major injuries.^1^ Tricuspid valve (TV) injuries resulting from motor vehicle collisions are exceedingly rare, and their diagnosis is often delayed due to the subtle and insidious onset of symptoms. The combination of tricuspid regurgitation (TR), a patent foramen ovale (PFO) or any interatrial shunt, and multiple orthopedic injuries significantly increases the risk of paradoxical embolic stroke in affected patients. While isolated TV surgery is generally reserved for cases involving right heart failure,^2^ earlier surgical intervention may be warranted in patients with a PFO to mitigate this elevated stroke risk.

## Case Presentation

A 64-year-old patient with no significant medical history presented to our institution as a trauma activation following a head-on motor vehicle collision with a Glasgow Coma Scale score of 14 (spontaneous eye opening, confused, and obeys commands). Physical examination revealed bilateral chest wall tenderness, abdominal tenderness, and apparent orthopedic injuries. A chest x-ray revealed a left-sided pneumothorax requiring chest tube placement in the emergency department. Initial imaging also identified a right vertebral artery dissection, a dens fracture, and multiple fractures involving the ribs, bilateral femurs, left radius and ulna, and right second to fifth metatarsals. Troponin was also elevated at 0.334.

**Figure 1:**
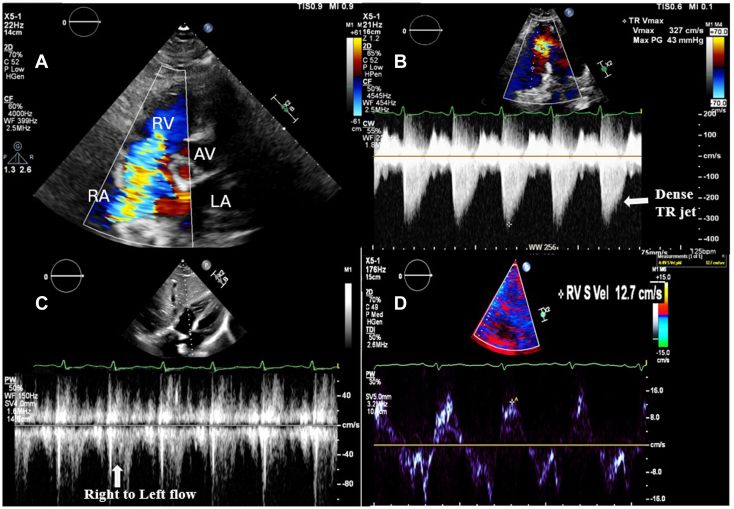
**(A)** Two-dimensional TTE, basal parasternal short-axis systolic view with color-flow Doppler, demonstrates severe TR; **(B)** continuous-wave spectral Doppler display demonstrates a triangle-shaped, dense profile (*arrow*) consistent with acute severe TR; **(C)** pulsed-wave spectral Doppler display of the interatrial septum demonstrates right-to-left Doppler flow profile (*arrow*); and **(D)** tissue Doppler spectral display of the tricuspid annulus demonstrates a myocardial systolic velocity profile, suggesting normal right ventricular function (12.7 cm/sec). *AV*, Aortic valve; *LA*, left atrium; *RA*, right atrium.

**Figure 2:**
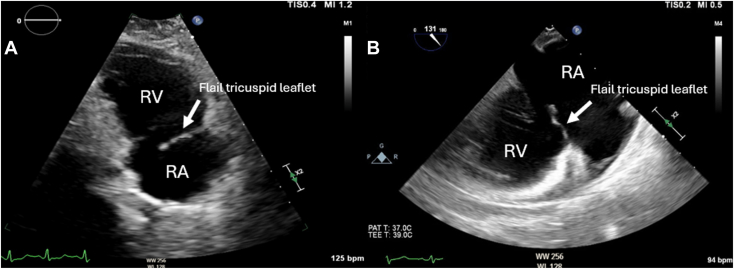
Follow-up two-dimensional TTE, apical right ventricular inflow **(A)**, and two-dimensional TEE, midesophageal modified bicaval long-axis (**B**; 131°) systolic views, demonstrate the flail anterior TV leaflet (*arrow*).

Upon admission to the intensive care unit, the patient became confused and hypoxic, with an oxygen saturation of 76% and respiratory rate of 34/min while on high-flow nasal cannula, necessitating intubation and mechanical ventilation. Brain magnetic resonance imaging revealed extensive multifocal punctate ischemic lesions in the cerebrum and cerebellum, and subsequent chest computed tomography showed scattered ground-glass opacities. Taken together with the patient's recent long bone fractures, these findings were suggestive of fat embolism syndrome. Transthoracic echocardiography (TTE) demonstrated severe TR (Figure 1, Video 1) and moderate pulmonary hypertension (right ventricular systolic pressure of 55 mm Hg). There was rapid equalization of right atrial and right ventricular pressures with normal right ventricular systolic function (S′, 12.7 cm/sec). The TTE also identified a significant right-to-left intracardiac shunt (Figure 1, Video 2). Transesophageal echocardiography (TEE) was attempted but unsuccessful due to cervical immobilization with a C-collar. Right heart catheterization was performed and estimated a Qp:Qs of 0.7. The decision was made to proceed with medical optimization prior to surgical intervention. The patient underwent operative fixation of their orthopedic injuries. With aggressive diuresis, their shunt fraction, which was estimated using a pulmonary artery catheter, improved. Follow-up TTE showed a flail TV leaflet with chordal rupture causing severe TR and a dilated right ventricle (RV) with normal systolic function (Figure 2A, Video 3).

**Figure 3:**
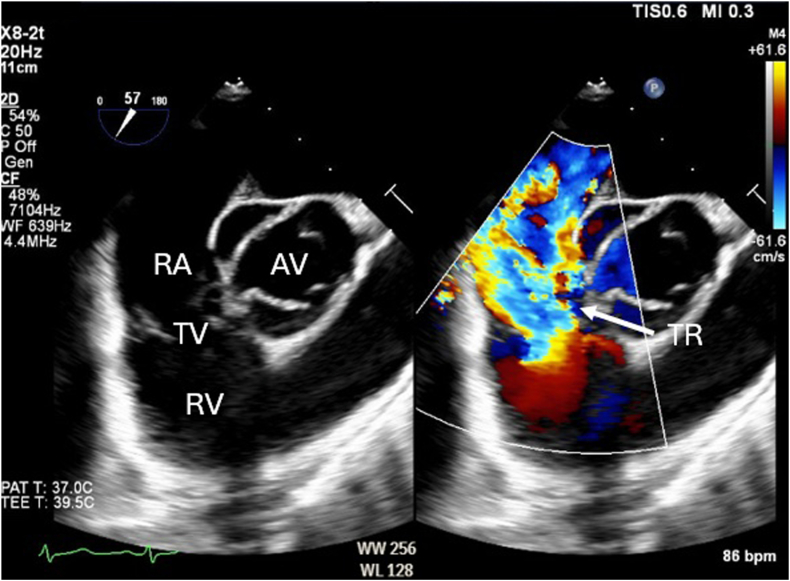
Two-dimensional TEE, midesophageal right ventricular inflow-outflow systolic view without (*left*) and with (*right*) color-flow Doppler, demonstrates severe TR and a possible interatrial shunt.

The patient was readmitted several months later for a TEE, which confirmed severe TR with poor leaflet coaptation due to a flail anterior TV leaflet from chordal rupture (Figures 2B and 3, Videos 4 and 5). A persistent right-to-left interatrial shunt was also noted (Video 6), and they were scheduled to undergo TV repair or replacement, PFO closure, and left atrial appendage excision.

**Figure 4:**
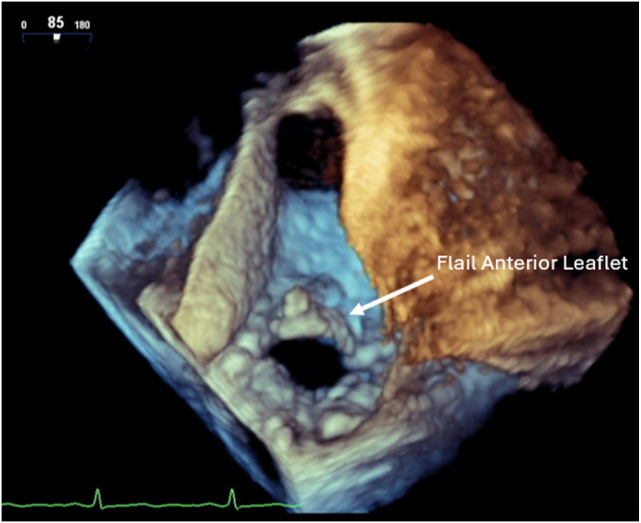
Three-dimensional TEE, midesophageal, volume-rendered short-axis systolic display from the ventricular perspective, demonstrates a flail anterior TV leaflet.

Initial intraoperative TEE redemonstrated normal global left ventricular systolic function, severe TR with a flail anterior leaflet and chordae rupture, large and redundant posterior leaflet, and right heart dilation (Figure 4, Videos 7 and 8). There was visually estimated moderate right ventricular systolic dysfunction. As repair of the valve was deemed difficult, the patient underwent a TV replacement with a 33 mm bioprosthetic valve, PFO closure, and left atrial appendage excision. Postbypass TTE showed trace central TR with no paravalvular leak. The mean gradient was 2 mm Hg.

**Figure 5:**
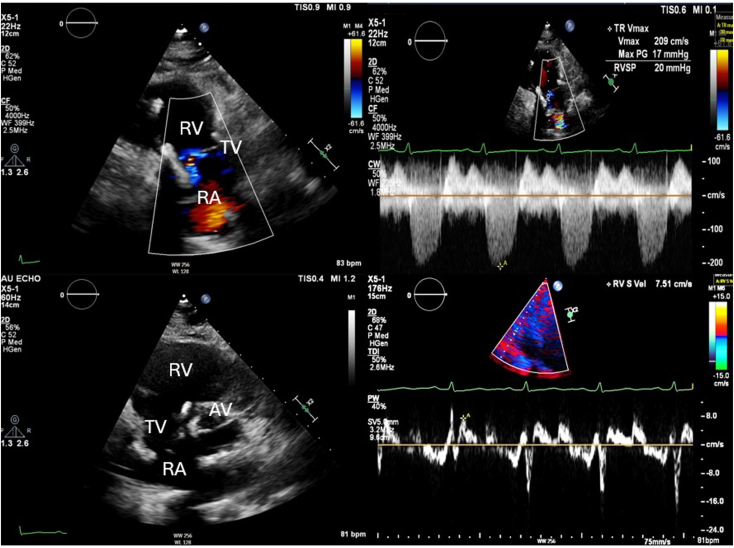
Postoperative two-dimensional TTE, parasternal long-axis right ventricular inflow systolic view with color-flow Doppler (*top left*) and continuous-wave Doppler (*top right*), right ventricular inflow-outflow short-axis systolic view (*bottom left*), and tissue Doppler spectral display of the tricuspid annulus (*bottom right*), demonstrates a dilated RV with moderate residual TR and decreased right ventricular systolic function.

Postoperative course was complicated by intermittent heart block, which spontaneously resolved. Follow-up TTE in 6 months showed a left ventricular ejection fraction of 50% to 55% with mild left ventricular dilation, right ventricular dilation, moderately reduced right ventricular systolic function, and moderate TR with a central jet (Figure 5, Videos 9 and 10).

## Discussion

Blunt chest trauma remains a significant cause of morbidity and mortality following motor vehicle collisions. Although valvular injuries are rare, when they do occur, the left-sided valves are more frequently affected due to higher intracardiac pressures on the left side of the heart.^3^ Traumatic TV injury, although less common, can have serious clinical consequences and should be included in the differential diagnosis of patients with blunt cardiac trauma. A PFO is present in approximately 25% of healthy adults and is typically asymptomatic.^4^ However, elevated right atrial pressure as seen in moderate or severe TR, especially when acute, can reverse the normal interatrial pressure gradient, resulting in a right-to-left flow across an interatrial shunt. This shunting can have significant clinical consequences, including hypoxemia, platypnea-orthodeoxia, and paradoxical embolism, which may manifest as myocardial infarction, embolic stroke, or other systemic embolic events.^5^ In this case, the patient developed ischemic strokes in both the cerebrum and cerebellum, likely due to paradoxical fat emboli traversing the right-to-left interatrial shunt.

Initial cardiac evaluation in blunt trauma commonly includes electrocardiography, chest radiography, and measurement of cardiac biomarkers, such as troponin. Echocardiographic assessment should follow in patients with abnormal findings or a mechanism suggestive of cardiac injury. Transthoracic echocardiography serves as an effective initial tool for assessing TV integrity. However, its utility may be limited in patients with chest wall injuries or poor acoustic windows. In such cases, or when TTE findings are inconclusive, TEE is recommended. When available, three-dimensional echocardiography provides enhanced visualization of all three TV leaflets and spatial orientation, offering critical anatomic detail to guide diagnosis and surgical planning.^6^ Additionally, any suspicion of valvular injury should prompt thorough evaluation for associated septal defects.

Traumatic TR encompasses a broad clinical spectrum, ranging from an incidental murmur to cardiogenic shock due to right ventricular failure. Despite its potential hemodynamic impact, traumatic TR is frequently underdiagnosed in the acute setting, particularly in polytrauma patients, where more overt injuries tend to overshadow subtle cardiac findings.^7^ As such, diagnosis is often delayed until signs of right heart failure or arrhythmias become evident. The mechanism of TV injury may be a result of direct trauma to the valve, compression of the right heart between the sternum and spine, and/or rupture of the subvalvular apparatus due to a sudden rise in right ventricular pressure during isovolumetric contraction against a closed TV.^7^ Chordal or papillary muscle rupture may present acutely, while papillary muscle injury may evolve more insidiously, beginning with myocardial contusion and progressing through inflammation, hemorrhage, and necrosis, eventually leading to delayed-onset TR.

**Figure 6:**
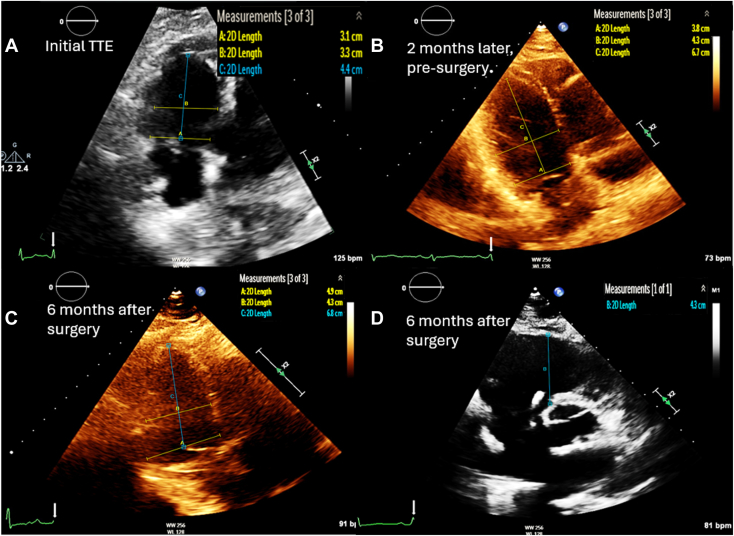
Serial two-dimensional TTE, obtained at baseline, 2 months, and 6 months postoperatively, right ventricular focused apical 4-chamber **(A**, **B** and **C)** and right ventricular short-axis inflow-outflow **(D)** systolic views, demonstrates progressive dilation of the RV leading to TV replacement surgery, with residual, persistent right ventricular dilation postoperatively.

The RV is a compliant structure that is more tolerant to volume overload than pressure overload.^8^ However, with chronic volume overload in severe TR, permanent right ventricular dilatation occurs, leading to increased tricuspid annular area and worsening TR, creating a vicious cycle of regurgitation and dilatation (Figure 6).^9^ Similarly, enlargement of the RV can lead to increased oxygen demand and eventual ischemia, further worsening right ventricular dysfunction. Due to the principle of interventricular interdependence, left ventricular diastolic filling is negatively affected by chronic right ventricular dilatation, leading to decreased left ventricular preload and cardiac output.^8^

Currently, there are no specific guidelines for the surgical management of traumatic TR, largely due to the rarity of these cases. As such, clinicians often extrapolate recommendations from the 2020 American Heart Association and American College of Cardiology guidelines, which provide class IIa evidence supporting isolated TV surgery in patients with right-sided heart failure and severe primary TR and class IIb evidence for surgery in asymptomatic patients with severe TR and progressive right ventricular dysfunction.^2^ Several studies suggest that intervention prior to the development of right heart failure can prevent or limit right atrial dilation and permanent arrhythmias and may allow for valve repair instead of replacement.^7^^,^^10^ When feasible, valve repair is generally preferred over replacement, particularly in younger patients, as it is associated with better outcomes and avoids the long-term risks associated with prosthetic valves.^11^ A large systematic review and meta-analysis found an association between right-to-left shunt via a PFO and cryptogenic stroke, potentially indicating a role for surgical intervention in patients with PFO and associated right-to-left shunt to mitigate the risk of paradoxical embolism.^12^ In our patient's case, a multidisciplinary review concluded that earlier surgical intervention would likely have mitigated right ventricular and left ventricular dilation and remodeling and would have also reduced the risk of paradoxical embolic stroke. Although there is no time frame distinguishing early from delayed repair, the optimal timing is when the diagnosis is established and the patient is clinically stable.

## Conclusion

Traumatic TV injury causing right-to-left flow across a PFO is exceedingly rare. This case underscores the importance of early recognition and timely surgical management of TR, particularly when accompanied by a PFO. Given the potential for irreversible right and left heart systolic dysfunction, persistent arrhythmias, and embolic events, clinicians should maintain a high index of suspicion and consider surgical intervention in appropriate cases.

## Ethics Statement

The authors declare that the work described has been carried out in accordance with The Code of Ethics of the World Medical Association (Declaration of Helsinki) for experiments involving humans.

## Consent Statement

The authors declare that since this was a non-interventional, retrospective, observational study utilizing de-identified data, informed consent was not required from the patient under an IRB exemption status.

## Supplementary Material

Video 1 Initial two-dimensional TTE, basal parasternal short-axis systolic view with color-flow Doppler, demonstrates severe TR.

Video 2 Two-dimensional TTE, subcostal view with color-flow Doppler, demonstrates a possible atrial septal defect with right-to-left flow.

Video 3 Two-dimensional TTE, apical right ventricular inflow view, demonstrates the flail TV leaflet.

Video 4 Two-dimensional TEE, midesophageal modified bicaval long-axis (131°) view, demonstrates the flail anterior TV leaflet.

Video 5 Two-dimensional TEE, midesophageal right ventricular inflow-outflow view without (*left*) and with (*right*) color-flow Doppler, demonstrates severe TR and a possible interatrial shunt.

Video 6 wo-dimensional TEE, midesophageal bicaval view with agitated saline contrast (bubble) study administered via the left superior vena cava, demonstrates an interatrial right-to-left shunt.

Video 7 Three-dimensional TEE, midesophageal, volume-rendered short-axis display from the ventricular perspective, demonstrates a flail anterior TV leaflet with ruptured chordae tendineae.

Video 8 Three-dimensional TEE, midesophageal volume-rendered short-axis display using photorealistic imaging from the ventricular perspective, demonstrates a flail anterior TV leaflet with ruptured chordae tendineae.

Video 9 Two-dimensional TTE, apical right ventricular inflow view, demonstrates a well-seated bioprosthetic TV with intravalvular, but no paravalvular, TR.

Video 10 Postoperative two-dimensional TTE, right ventricular inflow-outflow view, demonstrates a persistently dilated RV with reduced right ventricular systolic function.
